# Integrating Coronary Artery Assessment and Myocardial Late Enhancement Imaging With Photon-Counting Detector CT: Visualizing the Invisible

**DOI:** 10.1161/CIRCIMAGING.124.017238

**Published:** 2024-10-10

**Authors:** Marie-Julie D.K. Lemmens, Samuel Heuts, Elham Bidar, Joachim E. Wildberger, Casper Mihl, Martijn W. Smulders

**Affiliations:** 1 Cardiovascular Research Institute Maastricht, Maastricht University, the Netherlands (MJ.D.K.L., S.H., E.B., J.E.W., C.M., M.W.S.).; 2 Department of Radiology and Nuclear Medicine (MJ.D.K.L., J.E.W., C.M.), Maastricht University Medical Center+, the Netherlands.; 3 Department of Cardiology (MJ.D.K.L., M.W.S.), Maastricht University Medical Center+, the Netherlands.; 4 Department of Cardiothoracic Surgery (S.H., E.B.), Maastricht University Medical Center+, the Netherlands.

**Keywords:** coronary artery bypass, coronary artery disease, magnetic resonance imaging, myocardial infarction, tomography, x-ray computed

A 59-year-old female patient presented with dyspnea on exertion at the outpatient clinic. After an exercise test revealed unspecific ST-segment deviation, and the patient remained symptomatic despite medical therapy, further testing was considered. Invasive coronary angiography (ICA) revealed significant 2-vessel disease of the left anterior descending and left circumflex coronary arteries (Figure S1). Transthoracic echocardiography showed a decreased left ventricular ejection fraction, an aortic aneurysm, and mild-to-moderate aortic regurgitation of the tricuspid valve. To assess the complete aorta in more detail, preoperative contrast-enhanced computed tomography (CT) was performed and confirmed the presence of an isolated aortic root aneurysm (diameter 55×58 mm). Consequently, the patient was planned for aortic root replacement in combination with coronary artery bypass grafting.

During surgery, the aortic valve was sclerotic and retracted, and could not be preserved. As such, aortic root replacement with a stentless prosthesis was performed, alongside concomitant coronary artery bypass grafting (graft formula: left internal mammary artery to left anterior descending, and venous graft from aorta to obtuse marginal).

Postoperative electrocardiography (ECG) exams were unremarkable, showing sinus rhythm without ST-segment elevation. However, cardiac biomarkers were significantly increased 24 hours after surgery (Table S1). In the absence of ECG-abnormalities alongside unchanged echocardiographic findings, an ECG-gated CT angiography (CTA) of the coronary arteries and grafts was performed 3 days postoperatively, followed by a late enhancement (LE) scan 10 minutes after iodinated contrast media administration to evaluate the presence of possible myocardial infarction. The CTA and LE scan were acquired on a first-generation photon-counting detector (PCD) CT (NAEOTOM Alpha, Siemens Healthineers, Forchheim, Germany). The acquisition protocol and reconstruction parameters are presented in Table S2. This examination revealed a patent left internal mammary artery to left anterior descending graft (Figure 1A), while patency of the venous graft at the level of anastomosis with the obtuse marginal branch was impaired (Figures 1C and 2A). Interestingly, the LE scan already depicted focal subendocardial late iodine enhancement at the basal inferolateral wall (Figure 3C), strongly suggestive of myocardial infarction. Extracellular volume (ECV) measurements revealed a focal ECV increase in this region (Figures 2B, 3A, and 3B). The patient was discharged on the sixth postoperative day to the referring center for further recovery. Cardiac magnetic resonance (CMR) imaging was conducted 2 weeks later when the patient was stabilized. Cine imaging showed hypokinesia of the basal and mid inferolateral wall (Video S1), and LE-CMR visualized subendocardial late gadolinium hyperenhancement (<50% transmural). At this location, an increased T2 signal was observed, which- together with the aforementioned results- supports the diagnosis of periprocedural myocardial infarction (Figure 3D–3F). Subsequent ICA was performed and confirmed occlusion of the venous graft (Figure 1B and 1D; Figure S2).

**Figure 1.**
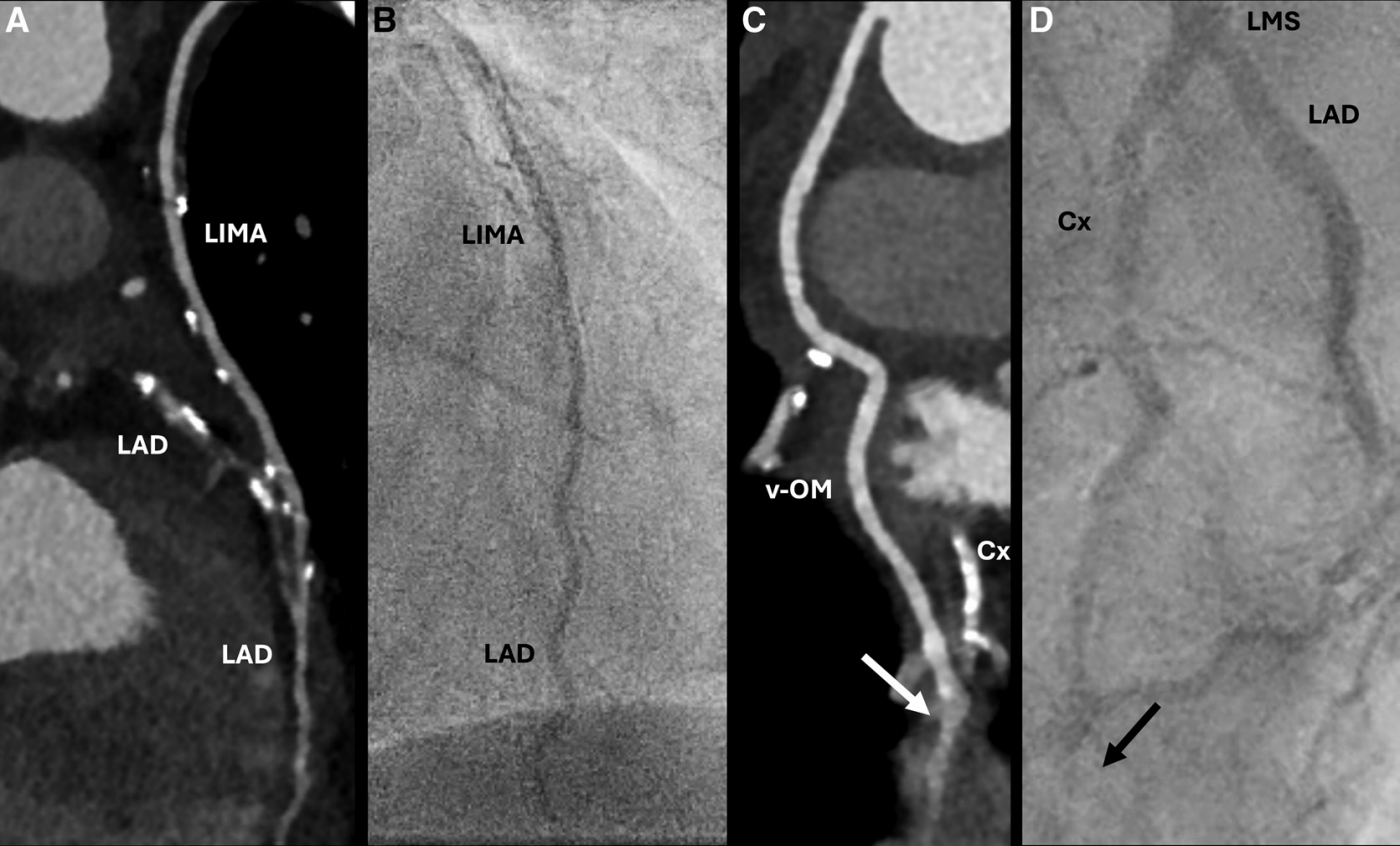
**Comparison between photon-counting detector computed tomography (PCD-CT) angiography and invasive coronary angiography (ICA) findings in the postoperative phase.** A multiplanar reformation (MPR) of the PCD-CT scan visualizes patency of the left internal mammary artery (LIMA) graft with anastomosis to the left anterior descending (LAD) coronary artery (**A**), confirmed by ICA (**B**). **C**, The MPR of the venous graft (v-OM) visualizes stenosis at the level of anastomosis (white arrow) and impaired filling of the distal left circumflex (Cx) coronary artery. **D**, ICA confirms impaired filling of the distal Cx and reveals complete occlusion of the venous graft, as it is not visible in the region of anastomosis (black arrow). LMS indicates left main stem coronary artery.

**Figure 2.**
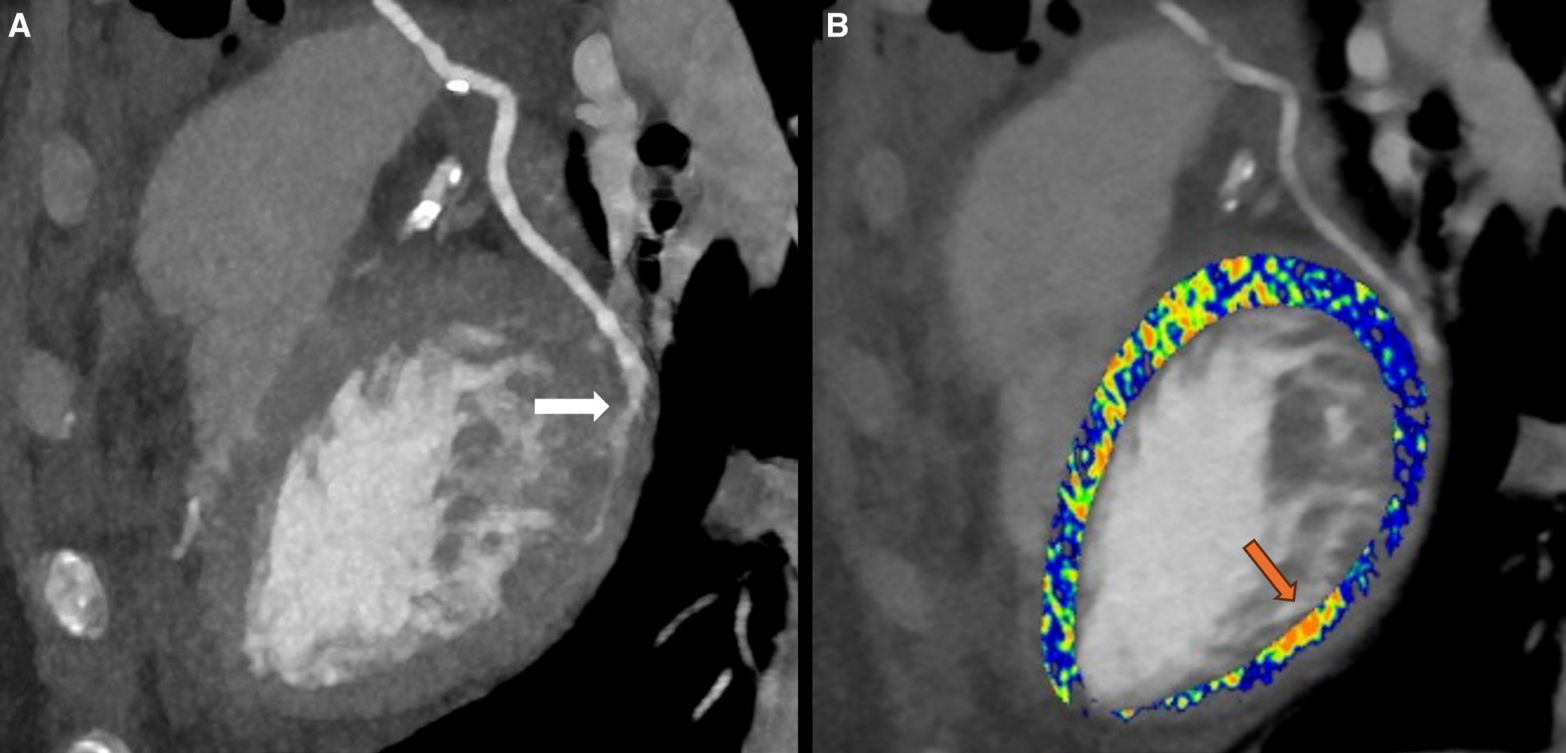
**Photon-counting detector computed tomography angiography of the venous graft from the aorta to the obtuse marginal branch of the left circumflex coronary artery with corresponding extracellular volume (ECV) map. A**, At the level of anastomosis (white arrow), stenosis of the venous graft is visualized and distal filling of the left circumflex coronary artery is impaired. **B**, A corresponding ECV map reveals focal ECV elevation (orange arrow) distal to the obstruction.

**Figure 3.**
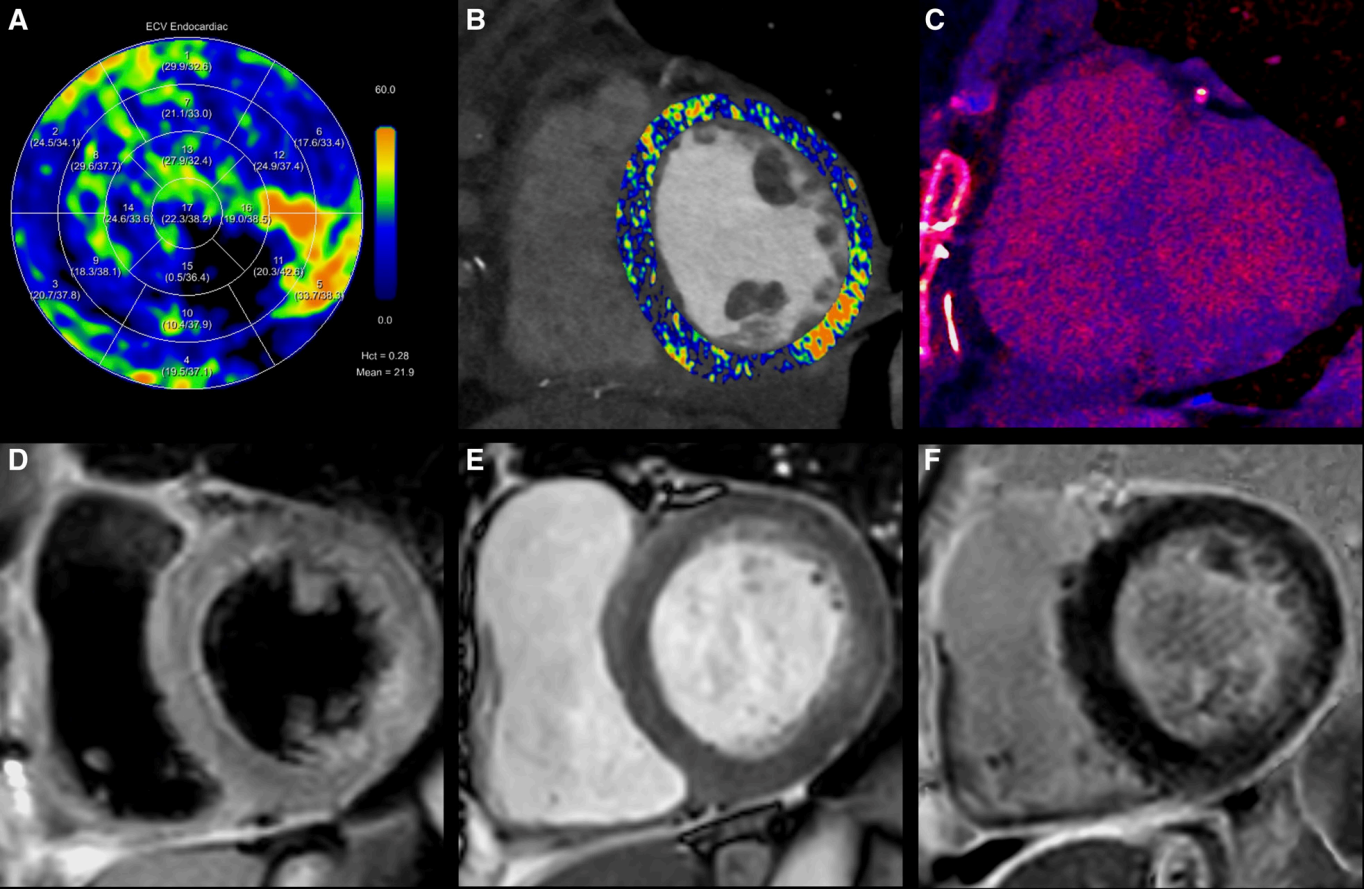
**Comparison between photon-counting detector computed tomography (PCD-CT) and cardiovascular magnetic resonance (CMR) imaging findings.** PCD-CT extracellular volume (ECV) maps show a focal ECV increase in the basal inferolateral segment (**A** and **B**), alongside late iodine enhancement on a calculated iodine map (**C**). CMR imaging validates these findings by providing the following results in the basal inferolateral segment: increased signal intensity on a T2-weighted sequence with fat suppression suggestive of edema (**D**), increased signal intensity and wall motion abnormalities on a short axis cine image after gadolinium administration (**E**), and late gadolinium enhancement (**F**).

At follow-up, the patient has fully resumed daily activities without symptoms of dyspnea or angina. A complete timeline of events is presented in Table S1.

Recently, PCD-CT has entered the field of cardiovascular imaging as a promising and innovative CT imaging technique. In this case report, we present the unique application of PCD-CT as a one-stop-shop modality, exploring its ability to conduct both coronary artery assessment and LE imaging in a postoperative patient following cardiac surgery.

LE is well-established within CMR imaging for the detection and assessment of myocardial fibrosis, while its application with conventional energy-integrating detector CT was limited due to a relatively low contrast-to-noise ratio.^1^ With its increased spatial and temporal resolution and inherent spectral features, PCD-CT might facilitate this concept and expand the role of CT in myocardial tissue assessment.^2^ In our postoperative patient, LE PCD-CT visualized a region of late iodine enhancement in the inferolateral myocardium, which exactly correlated with late gadolinium enhancement on CMR. The patency of the venous graft responsible for perfusion of this region was impaired on PCD-CTA, a finding later confirmed by ICA. We hypothesize that flow impairment due to stenosis at the level of the anastomosis resulted in progressive thrombotic occlusion of the graft as visualized with ICA ≈1.5 months later.

ECV measurements obtained through LE PCD-CT offer a quantitative assessment of an enlarged ECV fraction. A recent study has demonstrated the feasibility of PCD-CT ECV measurements compared with CMR in a cohort with different cardiac imaging indications.^3^ The use of a PCD system eliminates the need for noncontrast scanning to obtain iodine-specific images necessary for ECV calculation, which is a feature shared with dual-energy CT. Nonetheless, PCD systems are not limited by the same restrictions as dual-energy data generation with energy-integrating CT. In our case, an enlarged ECV was clearly visible representing ischemic changes within the myocardium after 3 days.

The current case offers several potential clinical applications for PCD-CTA and LE imaging as a one-stop-shop approach. In light of graft function assessment, graft failure may present as an acute coronary syndrome, for which ICA is generally indicated as it facilitates both diagnostic and therapeutic options. However, silent graft failure without evident functional or electrophysiological abnormalities, which can be present in up to 11% of venous grafts before hospital discharge, may present more subtle.^4^ Importantly, ICA carries the risk of coronary and vascular complications, particularly after coronary reimplantation following root surgery. As a result, routine ICA of graft patency following coronary artery bypass grafting is uncommon. CTA may be a valuable diagnostic tool in these instances and could guide therapy, with PCD systems enabling more precise coronary assessment with ultra-high-resolution scanning. This feature is particularly beneficial in the assessment of coronary grafts and anastomoses, which is typically affected by surgical clip and calcium artifacts. With respect to infarct detection, CMR is less appropriate for postoperative patients, who generally do not endure prolonged fixed positions or comply with breath-holding commands. In addition, these patients are often still dependent on CMR-incompatible devices, such as temporary epicardial pacing wires and continuous rhythm monitoring. As a combined procedure, PCD-CTA for graft function in conjunction with LE and ECV measurements is a straightforward approach to minimize periprocedural risks while yielding promising diagnostic results compared with sequential postoperative ICA and CMR, respectively.

In conclusion, this case illustrates the feasibility of incorporating LE imaging and ECV measurements in a multiphasic PCD-CT scan protocol evaluating both patency of grafts after coronary artery bypass grafting and coronary arteries, as well as functional assessment of the myocardium.

## Article Information

### Acknowledgments

Written informed consent was obtained before the preparation of this article.

### Sources of Funding

Dr Heuts was supported by the Dekker grant (Dutch Heart Foundation). Drs Mihl and Smulders were supported by an academic grant from the Maastricht University Medical Center+.

### Disclosures

Dr Wildberger received institutional grants from Clinical Trial Center Maastricht (Adora/Oldelft, Anaconda, Bard, Bayer, Bentley, Boston, Brainlab, GE, Inari Medical, Johnson & Johnson, Merit Medical, Nicolab, Philips, Sectra, Siemens, and Stryker) and is on the speaker’s bureau of Bayer and Siemens, all outside the submitted work. Dr Mihl is on the speaker’s bureau of Bayer Healthcare. Dr Smulders received speaker’s fee for ESC Highlights ’22 and ’23–Daiichi Sankyo Europe. The other authors report no conflicts.

### Supplemental Material

Tables S1 and S2

Figures S1 and S2

Video S1
